# Realizing high figure of merit in heavy-band *p*-type half-Heusler thermoelectric materials

**DOI:** 10.1038/ncomms9144

**Published:** 2015-09-02

**Authors:** Chenguang Fu, Shengqiang Bai, Yintu Liu, Yunshan Tang, Lidong Chen, Xinbing Zhao, Tiejun Zhu

**Affiliations:** 1 State Key Laboratory of Silicon Materials and School of Materials Science and Engineering, Zhejiang University, Hangzhou 310027, China; 2 State Key Laboratory of High Performance Ceramics and Superfine Microstructure, Shanghai Institute of Ceramics, Chinese Academy of Sciences, Shanghai 200050, China; 3 Key Laboratory of Advanced Materials and Applications for Batteries of Zhejiang Province, Zhejiang University, Hangzhou 310027, China

## Abstract

Solid-state thermoelectric technology offers a promising solution for converting waste heat to useful electrical power. Both high operating temperature and high figure of merit *zT* are desirable for high-efficiency thermoelectric power generation. Here we report a high *zT* of ∼1.5 at 1,200 K for the *p*-type FeNbSb heavy-band half-Heusler alloys. High content of heavier Hf dopant simultaneously optimizes the electrical power factor and suppresses thermal conductivity. Both the enhanced point-defect and electron–phonon scatterings contribute to a significant reduction in the lattice thermal conductivity. An eight couple prototype thermoelectric module exhibits a high conversion efficiency of 6.2% and a high power density of 2.2 W cm^−2^ at a temperature difference of 655 K. These findings highlight the optimization strategy for heavy-band thermoelectric materials and demonstrate a realistic prospect of high-temperature thermoelectric modules based on half-Heusler alloys with low cost, excellent mechanical robustness and stability.

## Abstract (web summary)

Thermoelectric materials could be used to convert waste heat into useful electricity, but the ideal substance needs to both optimize the electrical power factor and suppress thermal conductivity. Here, the authors report a high figure of merit of 1.5 at 1,200 K in the *p*-type half-Heusler alloy FeNbSb.

## Graphical abstract

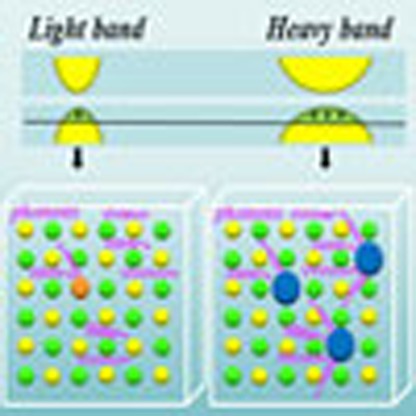

The demand for sustainable energies has sparked significant research into different types of energy conversion technologies in the past decades. Thermoelectric materials, which can directly convert waste heat into usable electricity, have received more and more attention for promising application in energy harvesting^1^,^2^. The conversion efficiency *η* of a thermoelectric device is limited by the Carnot efficiency *η*_c_, and the figure of merit *zT* of the thermoelectric materials, which is expressed as *zT*=α^2^σ*T*/(*κ*_e_+*κ*_L_), where *α*, *σ*, *T*, *κ*_e_ and *κ*_L_ are the Seebeck coefficient. respectively, the electrical conductivity, the absolute temperature and the electronic and lattice components of total thermal conductivity *κ* (ref. 1). Thus, a high *η*_c_ and a high *zT* will result in enhanced conversion efficiency. The thermoelectric parameters *α*, *σ*, and *κ*_e_ are intimately interrelated via carrier concentration and it has been a big challenge to decouple the thermal and electrical properties. Two main strategies, therefore, have been individually adopted to improve *zT*. One is to maximize the power factor *α*^2^*σ* through optimal doping and band engineering^1^,^3^,^4^. The other targets to reduce the lattice thermal conductivity *κ*_L_ by nanostructuring or phonon engineering^5^,^6^.

Traditional good thermoelectric materials, such as Bi_*x*_Sb_2-*x*_Te_3_ alloys near room temperature, PbTe_1−*x*_Se_*x*_ alloys at moderate temperature and Si_1−*x*_Ge_*x*_ alloys at high temperature, have high carrier mobility *μ* and reduced *κ*_L_ (refs 7, 8). A common character of these materials is that their band structures near the Fermi levels are dominated by the *s* or *p* electronic states, accounting for the low density of states effective mass m* and high *μ*. These light-band thermoelectric semiconductors with small m* (0.1 *m*_e_–1.0 *m*_e_) generally request relatively low-optimal carrier concentration *p*_opt_ (10^19^–10^20^ cm^−3^), as shown in Fig. 1a, a low content of dopants is enough to optimize their power factors.

In recent years, some other semiconductors have also been identified as promising high-performance thermoelectric materials, such as tin selenides^2^, filled skutterudites^9^ and half-Heusler compounds^10^,^11^. Most of them contain transition metal elements, such as Fe, Co, Ni *et al*., and their localized 3*d* states make the valence band maximum or conduction band minimum flat and heavy^12^,^13^. Typically, the m* of these heavy-band materials are in the range of 2 *m*_e_–10 *m*_e_ (Fig. 1a). Thus, higher carrier concentrations, which demands for higher contents of dopants, are necessary to optimize the power factors. For example, the *p*_opt_ of heavy-band ZrNiSn alloys is ∼4 × 10^20^ cm^−3^, one order of magnitude higher than that of PbTe (∼3 × 10^19^ cm^−3^), while the *p*_opt_ of filled CoSb_3_ and FeNbSb system with larger m* are above 10^21^ cm^−3^ (Fig. 1b). Note that even though these heavy-band thermoelectric materials have large m* and hence low *μ*, their optimal power factors are 2–3 times higher than the state-of-the-art light-band PbTe, which is an important reason making these heavy-band thermoelectrics promising for power generation. An immediate question arises that what is the effective optimization strategy for achieving high *zT* heavy-band thermoelectric materials?

Alloying (substitution or doping) creates point-defect scattering for phonons due to mass fluctuation and strain field fluctuation between the host atoms and alloying atoms^14^, and results in reduced *κ*_L_. In thermoelectric materials, dopants not only supply carriers to optimize the power factor, but deduce point-defect scattering of phonons to suppress κ_L_. For light-band thermoelectric semiconductors, the *p*_opt_ is relatively low and a slight content of dopants are enough to optimize the power factor^15^,^16^, and the dopants usually contribute less to the *κ*_L_ reduction. By contrast, in heavy-band semiconductors, higher contents of dopants are demanded for optimizing the carrier concentration to reach the same Femi level (Fig. 1c). For example, ∼20% Sn was doped to optimize the power factor of heavy-band ZrCoSb compounds^17^. Such a high content of dopant will also definitely create strong point-defect phonon scattering to reduce *κ*_L_. Furthermore, stronger point-defect phonon scattering may occur if the doping atoms have larger mass and strain field fluctuations compared with the host atoms (Fig. 1c), which could be an effective strategy for simultaneously optimizing electrical power factor and reducing thermal conductivity in heavy-band thermoelectric materials.

A high Carnot limit, *η*_c_=(*T*_H_−*T*_C_)/*T*_H_, needs a large temperature difference between the temperature of hot side, *T*_H_, and temperature of cold side, *T*_C_, of the thermoelectric device. Therefore, high temperature thermoelectric materials with superior properties are highly desirable for power generation operating above 1,000 K. Half-Heusler compounds have attracted more and more attention due to their good electrical and mechanical properties and thermal stability at high temperatures^11^,^17^,^18^,^19^,^20^,^21^,^22^,^23^,^24^,^25^,^26^. The highest *zT*s of ∼1.0 have been reported for *n*-type ZrNiSn-based half-Heusler alloys^18^,^20^,^21^,^24^. But developing high-performance *p*-type Zr-based half-Heusler compounds is still a big challenge^17^,^24^. Recently, we found that *p*-type Fe(V,Nb)Sb-based heavy-band half-Heusler compounds show great potential as high-temperature thermoelectric materials and a high *zT* of 1.1 has been reached at 1,100 K in FeNb_1−*x*_Ti_*x*_Sb with high Ti content up to 20%^19^,^27^. Although the *κ*_L_ of Ti-doped FeNbSb is remarkably reduced due to the enhanced point-defect scattering, it is still ∼3 times as high as the calculated minimum *κ*_L_ (∼1 W m^−1^ K^−1^)^19^. To achieve higher *zT* in *p*-type FeNbSb, it is imperative to further suppress its *κ*_L_. Based on the above consideration and Fig. 1c, selecting the high contents of doping atoms having larger mass and radius differences with the host atoms may lead to further *κ*_L_ reduction at optimal carrier concentration and hence enhanced *zT*.

Here we indeed demonstrate that the thermoelectric properties of *p*-type FeNbSb half-Heusler compound can be significantly enhanced through heavier Hf doping. A record-high *zT* of up to 1.5 at 1,200 K has been obtained in the heavy-band FeNb_1−*x*_Hf_*x*_Sb alloys. High contents of Hf and Zr dopants result in enhanced point-defect scattering of phonons, and the Hf doping at Nb site leads to the stronger phonon scattering. Interestingly, the electron–phonon scattering is found to also strongly contribute to the reduced *κ*_L_ at high dopant contents. An eight *n–p* couples prototype half-Heusler thermoelectric module, based on our high-performance *n*-type ZrNiSn (ref. 18) and *p*-type FeNbSb compounds, is successfully assembled for the first time in this work. A maximum conversion efficiency of 6.2% and a power density of 2.2 W cm^−2^ under a temperature difference of 655 K are achieved, exhibiting the great potential of low-cost *p*-type FeNbSb half-Heusler compounds for high temperature power generation.

## Results

### *zT* enhancement and prototype half-Heusler module

High-quality FeNb_1−*x*_Hf_*x*_Sb and FeNb_1−*y*_Zr_*y*_Sb (*x*, *y*=0–0.16) samples were fabricated by levitation melting and spark plasma sintering. X-ray diffraction (XRD) patterns show that the single phase products were obtained (Supplementary Fig. 1). Figure 2a shows the *zT* values of these samples. A peak *zT* of ∼1.5 is reached at 1,200 K for FeNb_0.88_Hf_0.12_Sb and FeNb_0.86_Hf_0.14_Sb, ∼40% higher than that of Ti-doped FeNbSb^19^, and the *zT*s are remarkably higher than other well-known state-of-the-art *p*-type high-temperature thermoelectric materials over the whole temperature range. As known, the average *zT*_avg_ is more important than the peak *zT* for thermoelectric device application. The *zT*_avg_ of FeNb_0.88_Hf_0.12_Sb sample is calculated to be ∼0.8 and ∼1.0 in the temperature range of 300–1,200 and 500–1,200 K, respectively, even exceeding the industry benchmark set by conventional *p*-type SiGe alloys (peak *zT*=0.6)^17^.

To corroborate the present results, the prototype high-temperature thermoelectric modules with eight *n–p* half-Heusler couples were assembled (Fig. 2b) for the first time based on the best *n*-type ZrNiSn-based alloys (thermoelectric properties are shown in Supplementary Fig. 2) and *p*-type FeNbSb compounds. The dimensions of the thermoelectric module made from the half-Heusler legs are 20 mm by 20 mm by 10 mm thick. Under conditions of hot/cold-side temperatures of 991 K/336 K, the half-Heusler module exhibited a maximum power output of 8.9 W and 6.2% conversion efficiency, which is significantly higher than the conversion efficiency of 4.5% for the commercial half-Heusler modules based on *n*-type ZrNiSn and *p*-type ZrCoSb-based half-Heusler alloys. Extrapolated values indicate that 8.1% is achievable when the hot-side temperature is up to 1,200 K. The calculated total area power density for this half-Heusler module is about 2.2 W cm^−2^, which is significantly higher than other thermoelectric modules^28^,^29^,^30^ (Supplementary Table 1). The theoretical conversion efficiency is also calculated for comparison (dash line in Fig. 2b), which is higher than the experimental value. The discrepancy could be due to the matching between *n*-type and *p*-type legs, the insufficient contacting and the large radiation and convection losses and insufficient accuracy of measurement. Especially, the contact resistance contributes to about 3.2% efficiency loss (Supplementary Discussion). More work is needed to improve the contacting electrical and thermal resistance and use thermal isolation between the half-Heusler legs.

### Decoupling of electrical and thermal properties

Why do the *p*-type heavy-band FeNb_1−*x*_Hf_*x*_Sb alloys have so high *zT*s? The thermoelectric properties of FeNb_1−*x*_Hf_*x*_Sb and FeNb_1−*y*_Zr_*y*_Sb compounds are presented in Fig. 3, and analysed by using the single parabolic band (SPB) model^31^,^32^. The samples are heavily doped and the hole concentration is almost independent of temperature before intrinsic excitation (Supplementary Fig. 3). The electrical conductivity *σ* of the FeNb_1−*x*_Hf_*x*_Sb and FeNb_1−*y*_Zr_*y*_Sb samples shows a metal-like behaviour and follows a temperature dependence of *T*^*−*1.5^ (Fig. 3a), implying an acoustic phonon-scattering-dominated charge transport. The Seebeck coefficient *α* decreases with increasing carrier concentration (Fig. 3b). The calculated *α* by the SPB model agrees well with the experimental data before the intrinsic excitation. The m* was estimated to be ∼6.9 *m*_e_ and almost unchanged at 300 and 800 K, as shown in the Pisarenko plot of Fig. 3c, indicating that the valence band structure has weak dependence on temperature and the dopant type of Hf, Zr and Ti.

**Figure 1:**
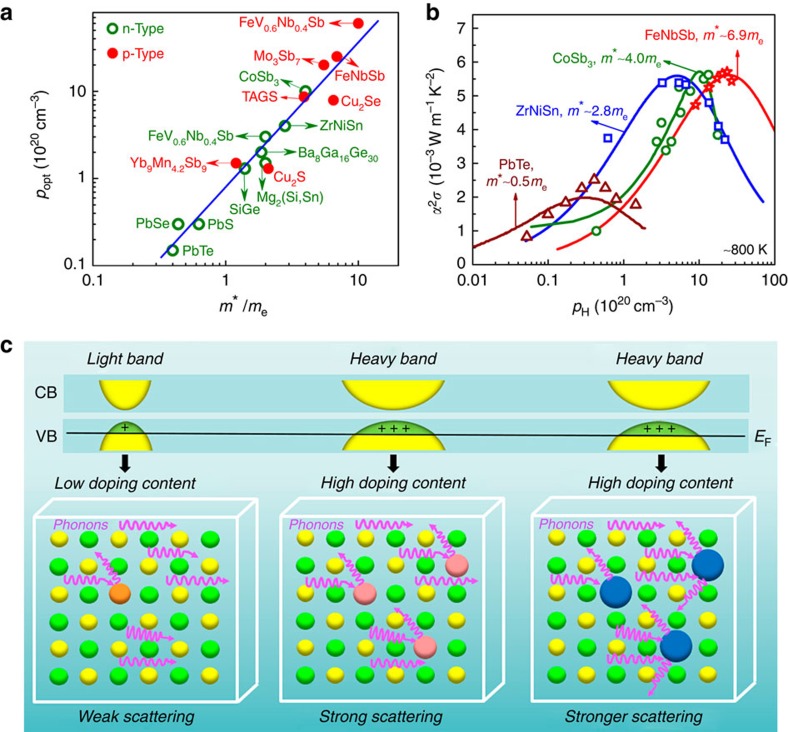
Comparison of transport character of light-band and heavy-band thermoelectric materials. (**a**) The optimal carrier concentration *p*_opt_ versus the density of state effective mass m* for thermoelectric materials^15^,^16^,^27^,^31^,^32^,^33^,^36^,^39^,^40^,^41^,^42^,^43^,^44^,^45^. The solid line is a guide for eyes. (**b**) Carrier concentration dependence of power factor for the typical light-band PbTe^15^, and the heavy-band system: *n*-type ZrNiSn^33^, *n*-type filled CoSb_3_^46^ and *p*-type FeNbSb near 800 K. (**c**) The schematic drawing shows the effect of band structure character on optimal doping content and hence phonon scattering.

**Figure 2:**
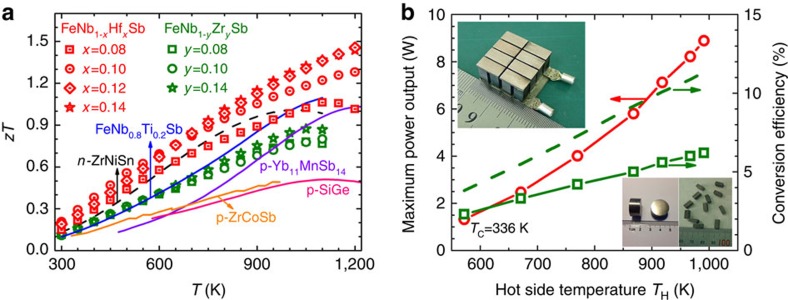
Thermoelectric performance for *p*-type FeNbSb-based HH compounds and prototype module. (**a**) *zT* comparison for Hf or Zr doped FeNbSb and other typical high temperature *p*-type thermoelectric materials^17^,^18^,^19^,^40^,^47^. (**b**) Maximum power output and conversion efficiency as a function of hot side temperature *T*_H_ for the thermoelectric device made from our best *n*-type ZrNiSn-based alloys and *p*-type FeNbSb HH compounds. The dash line represents the theoretical conversion efficiency of the module with a maximum value of 11.3%, assuming no electrical and thermal contact resistances.

**Figure 3:**
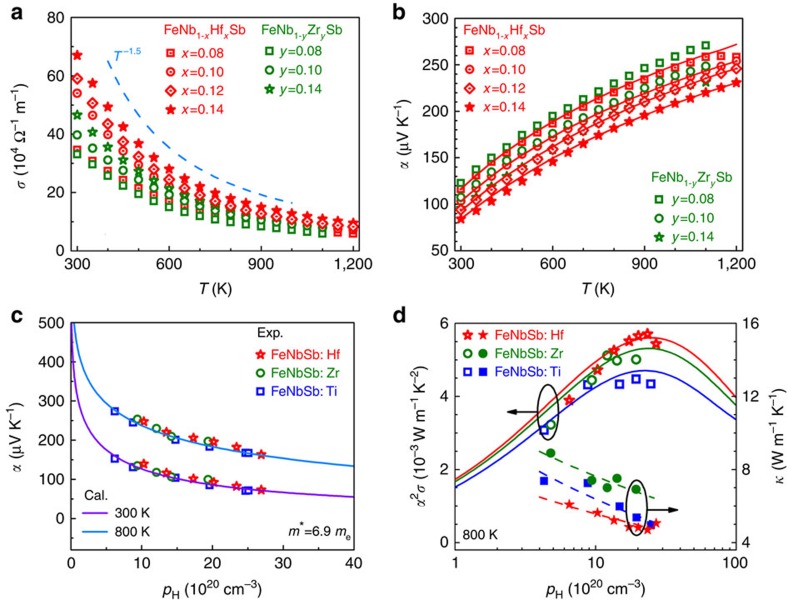
Thermoelectric properties for FeNb_1−*x*_Hf_*x*_Sb and FeNb_1−*y*_Zr_*y*_Sb samples. (**a**) Electrical conductivity *σ*. (**b**) Seebeck coefficient *α*. The *α* (**c**) and power factor *α*^2^*σ* and thermal conductivity (**d**) of Hf- and Zr-doped FeNbSb as a function of carrier concentration, together with the data for Ti-doped FeNbSb^19^. The solid lines in **b**–**d** were calculated by the SPB model.

The carrier concentration dependence of power factor for Hf- and Zr-doped FeNbSb samples at 800 K is shown in Fig. 3d, together with Ti doping data^19^. The optimal power factor ranges from 4.3 to 5.5 × 10^−3^ W m^−1^ K^−2^ at *p*_opt_ of ∼2 × 10^21^ cm^−3^, which are relatively high values among established thermoelectric materials and comparable to the optimized *n*-type ZrNiSn-based half-Heusler compounds^33^. Figure 3d also indicates that the power factors of Hf-doped FeNbSb are higher than that of Zr- or Ti-doped samples. Further analysis shows that the Hf dopant is more efficient in supplying carriers than Zr and Ti (Supplementary Fig. 4). Thus at the carrier concentration of ∼2 × 10^21^ cm^−3^ for *p*-type FeNbSb, the doping content of Hf, Zr and Ti is about 12, 14 and 16%, respectively (Supplementary Fig. 4a). The corresponding room temperature carrier mobility for these samples are 18.4, 15.0 and 13.8 cm^2^ V^−1^ s^−1^, indicating that the less doping content for Hf-doped FeNbSb is beneficial for relatively higher carrier mobility due to the reduced alloy scattering of carriers. Therefore, at the same carrier concentration, the Hf-doped FeNbSb has higher power factors than Zr- and Ti-doped samples (Supplementary Fig. 4b). It is noteworthy that the different dopants also generate different effects on the thermal conductivity (Fig. 3d). The heavier Hf dopant leads to the ∼30% lower thermal conductivity compared with the Zr dopant, consistent with the discussion relevant to Fig. 1c.

### Reduced lattice thermal conductivity and mechanisms

**Figure 4:**
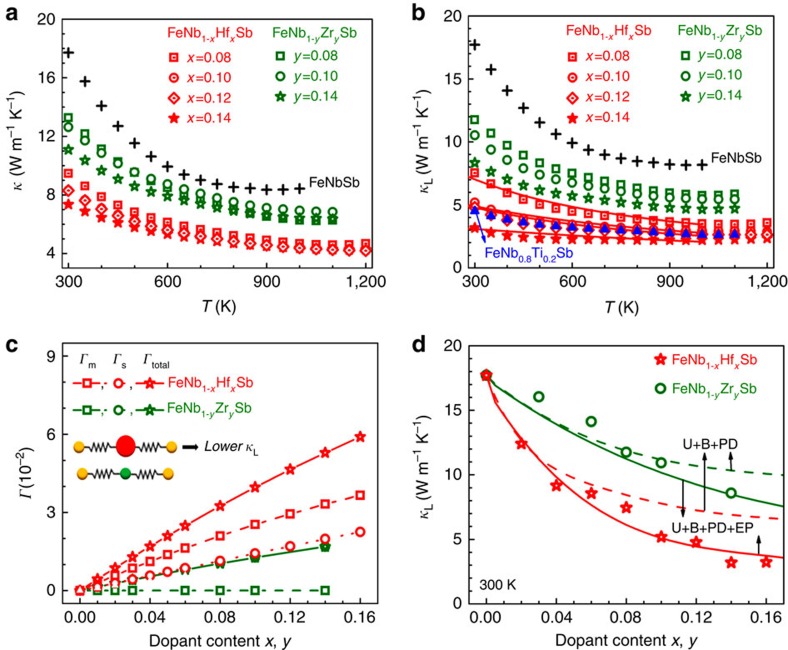
Thermal conductivity for FeNb_1−*x*_Hf_*x*_Sb and FeNb_1−*y*_Zr_*y*_Sb samples. (**a**) Total thermal conductivity *κ*. (**b**) Lattice thermal conductivity *κ*_L_. The solid curves in **b** are calculated using the Callaway model^36^,^37^. For comparison, *κ*_L_ of Ti-doped FeNbSb is also shown^19^. (**c**) The calculated disorder parameter *Γ* for the samples, where *Γ*_m_ (square) and *Γ*_s_ (circle) are mass and strain field fluctuation disorder parameters, respectively.^14^,^34^ *Γ*_total_=*Γ*_m_+*Γ*_s_. (**d**) Comparison of experimental and calculated *κ*_L_ for the samples at 300 K. The dash and solid curves are calculated without and with electron-phonon scattering, respectively. U, B, PD and EP denote the phonon-phonon Umklapp process, boundary, point-defect and electron-phonon scattering, respectively.

The temperature dependences of *κ* and *κ*_L_ of FeNb_1−*x*_Hf_*x*_Sb and FeNb_1−*y*_Zr_*y*_Sb compounds are presented in Fig. 4. The *κ*_L_ was obtained by subtracting the electronic component *κ*_e_ from the total thermal conductivity *κ*. *κ*_e_ was calculated via Wiedemann–Franz relationship *κ*_e_=*LσT*, where *L* is the Lorenz number determined under the SPB approximation^32^. Figure 4a shows the *κ* of Hf- and Zr-doped FeNbSb compounds are lower than that of FeNbSb. The decrease in *κ* mainly results from the greatly suppressed *κ*_L_. As shown in Fig. 4b, with the same doping content, the *κ*_L_ of Hf-doped FeNbSb is lower than that of Zr- and Ti-doped samples, and the high-temperature *κ*_L_ of FeNb_0.8_Ti_0.2_Sb is only close to that of FeNb_0.9_Hf_0.1_Sb, suggesting that Hf dopant leads to significantly reduced *κ*_L_ in FeNbSb even at a low content. The *κ*_L_ of FeNb_1−*x*_Hf_*x*_Sb decreases greatly with increasing Hf content. Especially, at 300 and 1,000 K the *κ*_L_ of FeNb_0.86_Hf_0.14_Sb has ∼80% and ∼70% reduction respectively, compared with that of FeNbSb, which is a key to the high *zT* in this composition.

Why is Hf dopant more efficient in suppressing *κ*_L_ of FeNbSb despite of lower optimal content? As aforementioned, high content of dopants will create strong point-defect scattering of phonons, leading to the suppressed *κ*_L_. Hf doping at Nb sites will deduce more remarkable point-defect scattering than Zr and Ti because of the larger mass and radius differences between Hf and Nb. For comparison, Fig. 4c presents the calculated disorder parameter *Γ* (larger *Γ* indicates stronger point-defect scattering of phonons^14^,^34^,^35^) for Hf and Zr at Nb sites, which obviously shows that the Hf creates stronger mass and strain field fluctuations, leading to lower *κ*_L_ in FeNb_1−*x*_Hf_*x*_Sb.

The *κ*_L_ of the samples was further calculated by the Callaway model^19^,^36^,^37^. Phonon–phonon Umklapp process, grain boundary and point-defect scattering of phonons were firstly considered in the modelling. At low doping content, the calculated *κ*_L_ has a good agreement with the experimental results (Fig. 4d). However, at high doping contents, the calculated *κ*_L_ significantly deviates from the experimentally values, suggesting that some other scattering sources should also contribute to the reduced *κ*_L_ at high Hf or Zr contents. With increasing dopant content, the carrier concentration largely increases up to 10^21^ cm^−3^. The electron–phonon interaction, an important part to scatter phonons in narrow semiconductors^38^, may exist in the *p*-type FeNbSb heavy-band system. With the electron–phonon scattering evolved, a good agreement between the experimental data and the calculated curves is reached (Fig. 4d). To corroborate this result, temperature dependence of *κ*_L_ was calculated for FeNb_1−*x*_Hf_*x*_Sb samples, and there is a good consistency with the experimental *κ*_L_ (Fig. 4b), indicating the enhanced electron–phonon scattering of phonons also contributes to the reduced *κ*_L_ for FeNb_1−*x*_Hf_*x*_Sb and FeNb_1−*y*_Zr_*y*_Sb, especially at high doping contents. The similar phenomenon is also found in other thermoelectric materials^36^. Thus the simultaneously enhanced point-defect and electron–phonon scattering of phonons concurrently contribute to the reduced *κ*_L_ in the heavy-band FeNb_1−*x*_Hf_*x*_Sb system.

## Discussion

In summary, by rationally selecting the heavier dopants at high contents, the interrelated thermoelectric parameters can be decoupled and the simultaneous optimization of electrical power factor and significant reduction in thermal conductivity can be achieved in heavy-band thermoelectric materials. Record-high *zT* of 1.5 in *p*-type FeNb_1−*x*_Hf_*x*_Sb heavy-band half-Heusler compounds demonstrates the effective optimization strategy for achieving high thermoelectric performance. A prototype thermoelectric module made of *n*-type ZrNiSn-based alloys and *p*-type FeNbSb compounds exhibits a high conversion efficiency of 6.2% and a high power density of 2.2 W cm^−2^ at a temperature difference of 655 K. These findings highlight the realistic prospect of high-temperature thermoelectric modules based on half-Heusler alloys with low cost, excellent mechanical properties and stability.

## Methods

### Synthesis

The ingots with nominal composition FeNb_1−*x*_Hf_*x*_Sb and FeNb_1−*y*_Zr_*y*_Sb (*x*, *y*=0–0.16) were prepared by levitation melting of stoichiometric amount of Fe (piece, 99.97%), Nb (foil, 99.8%), Hf (piece, 99.99%), Zr (foil, 99.99%) and Sb (block, 99.999%) under an argon atmosphere for several minutes. The ingots were remelted for four times to ensure homogeneity. The obtained ingots were mechanically milled (Mixer Mill MM200, Retsch) for 4 h under argon protection. The obtained powders were loaded into the graphite die and compacted by spark plasma sintering (SPS-1050, Sumitomo Coal Mining Co.) at 1,123 K for 10 min under 65 MPa in vacuum. The as-sintered samples, of which the relative densities were found to be ∼95%, were annealed at 1,073 K for 3 days.

### Characterization

Phase structures of the samples were investigated by XRD on a RigakuD/MAX-2550PC diffractometer using Cu K_α_ radiation (*λ*_0_=1.5406 Å). The XRD patterns of FeNb_1−*x*_Hf_*x*_Sb and FeNb_1−*y*_Zr_*y*_Sb show a single phase that can be indexed to the half-Heusler phase with a cubic MgAgAs-type crystal structure (space group, F43m) as shown in Supplementary Fig. 1. The lattice parameter of the samples increases with increasing dopant content as shown in Supplementary Fig. 5. The chemical compositions were checked by electron probe microanalysis (EPMA, JEOL and JXA-8100), which show that the actual compositions are close to the nominal ones (Supplementary Table 1). Scanning electron microscope and energy dispersive X-ray spectroscopy mapping were used to characterize the phase and compositional homogeneity (Supplementary Fig. 6). The average grain size of the sample was determined to be ∼0.8 μm from the transmission electron microscope (FEI, Tecnai G2 F30 S-Twin) image (Supplementary Fig. 6).

### Measurements

The Seebeck coefficient and electrical conductivity from 300 to 1,200 K were measured on a commercial Linseis LSR-3 system using a differential voltage/temperature technique and a d.c. four-probe method. The accuracy is ±5% and ±3%, respectively. The thermal conductivity *κ* was calculated by using *κ*=*DρC*_p_, where *ρ* is the sample density estimated by the Archimedes method. The thermal diffusivity *D* and specific heat *C*_p_ were measured by a laser flash method on Netzsch LFA457 instrument with a Pyroceram standard (Supplementary Fig. 7). The accuracy is ±3% and ±5%, respectively. The low-temperature Hall coefficients from 20 to 300 K were measured using a Mini Cryogen Free Measurement System (Cryogenic Limited, UK). The carrier concentration *p*_H_ was calculated by *p*_H_=1/(*eR*_H_), where *e* is the unit charge and *R*_H_ is the Hall coefficient. The estimated error of Hall coefficient is within ±10%. The carriers mobility *μ*_H_ was calculated by *μ*_H_=*σR*_H_. The samples with highest *zT* were repeatedly measured in Zhejiang University and Shanghai Institute of Ceramics, Chinese Academy of Science, and the results show good consistency (Supplementary Fig. 8). The high-temperature thermal stability of the sample was checked through the thermogravimetric analysis (Supplementary Fig. 9) and the accuracy is 5%.

### Thermoelectric module

For the eight *n–p* couple prototype module assembly, the cylindrical half-Heusler pucks were diced into legs of square 4 mm by 4 mm. Then the *n*-type and *p*-type half-Heusler legs were connected to metallic interconnects using high-temperature braze. The modules contain a total of 16 legs joined into 8 *n–p* couples, all connected electrically in series and thermally in parallel. The power output, internal resistance and energy conversion efficiency of the half-Heusler prototype modules were evaluated in vacuum by using PEM-2 testing system (ULVAC-RIKO, Inc.). The electrodes coexist stably with *p*/*n* half-Heusler alloys in the module's working temperature range from 300 to 1,000 K. The accuracy of measurement for output power and conversion efficiency is about 10–15%.

## Additional information

**How to cite this article:** Fu, C. *et al*. Realizing high figure of merit in heavy-band *p*-type half-Heusler thermoelectric materials. *Nat. Commun.* 6:8144 doi: 10.1038/ncomms9144 (2015).

## Supplementary Material

Supplementary Information Supplementary Figures 1-12, Supplementary Tables 1-2, Supplementary Discussion and Supplementary References
