# Supplementary material for: Realizing high figure of merit in heavy-band p-type half-Heusler thermoelectric materials
Source: Nat Commun. 2015 Sep 2;6:8144. doi: 10.1038/ncomms9144 (PMC4569725; doi:10.1038/ncomms9144)
Supplement: Supplementary Information — Supplementary Figures 1-12, Supplementary Tables 1-2, Supplementary Discussion and Supplementary References [file ncomms9144-s1.pdf]

Supplementary Figures

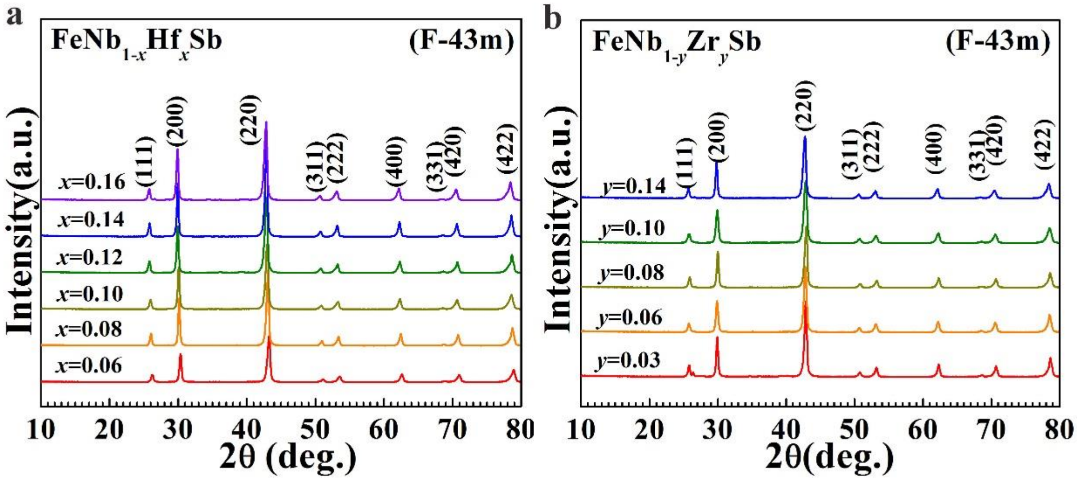

Supplementary Figure 1. XRD patterns for the $FeNb_{1-x}Hf_xSb$ (a) and $FeNb_{1-y}Zr_ySb$ samples (b). All the diffraction peaks can be indexed to the HH phase with a cubic MgAgAs-type crystal structure.

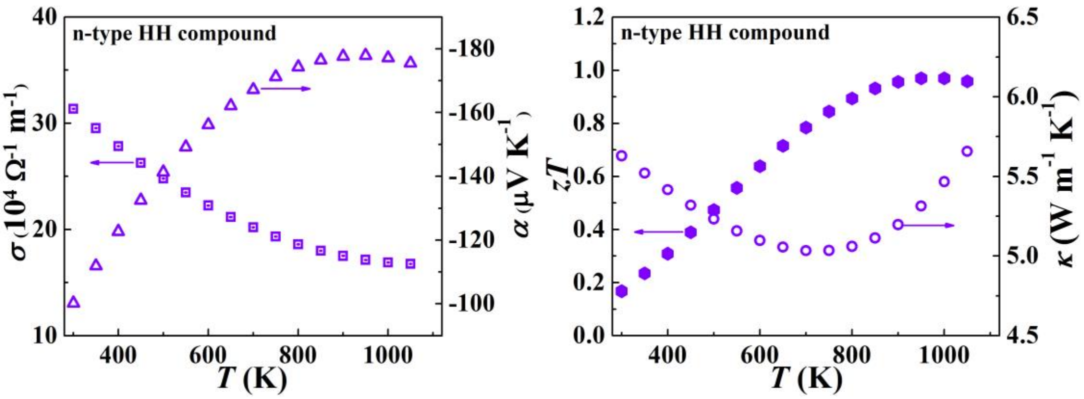

Supplementary Figure 2. Thermoelectric properties of the *n*-type half-Heusler compound, which was used for assembling the half-Heusler module.

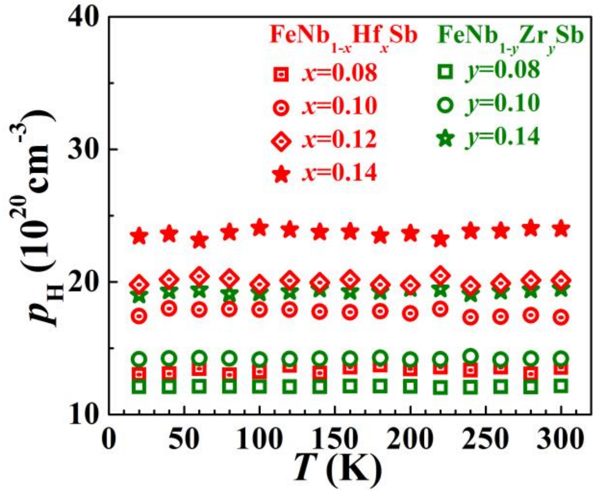

Supplementary Figure 3. Hall carrier concentration of $FeNb_{1-x}Hf_xSb$ and $FeNb_{1-y}Zr_ySb$ samples as a function of temperature. The almost temperature independent of carrier concentration indicates a heavily doped semiconductor character in these samples.

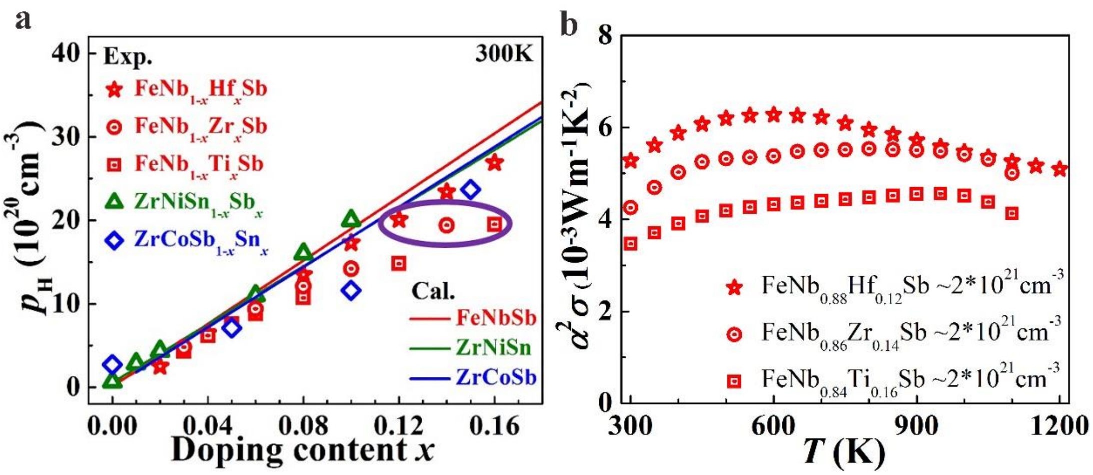

Supplementary Figure 4. a, Room carrier concentration versus doping content for FeNbSb, ZrNiSn[1] and ZrCoSb[2] HH compounds. The lines were calculated assuming that each doping atom supplies exactly one carrier. **b**, Power factor comparison for Hf, Zr, Ti doped FeNbSb samples under the similar carrier concentration of ~2×10^{21}cm^{-3} (purple ellipse in **a**). As shown in **a**, Hf doping in FeNbSb is more efficient to supply carriers compared with that of Zr/Ti dopants, but the carrier concentrations of all the doped FeNbSb samples are smaller than the calculated values. Similar phenomenon is also found for Sn doped ZrCoSb HH system[2]. However, for *n*-type ZrNiSn HH compound, Sb dopant is highly efficient to provides electrons[1]. Therefore, for HH compounds, in order to optimize their electrical properties, the doping efficacy of dopants should be carefully considered.

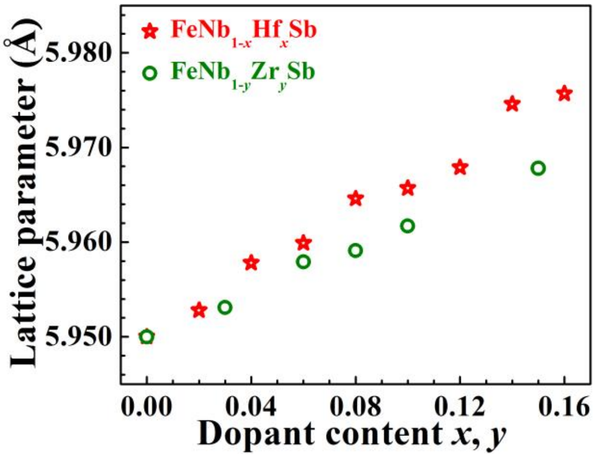

Supplementary Figure 5. Lattice parameter of $FeNb_{1-x}Hf_xSb$ and $FeNb_{1-y}Zr_ySb$ as a function of dopant content.

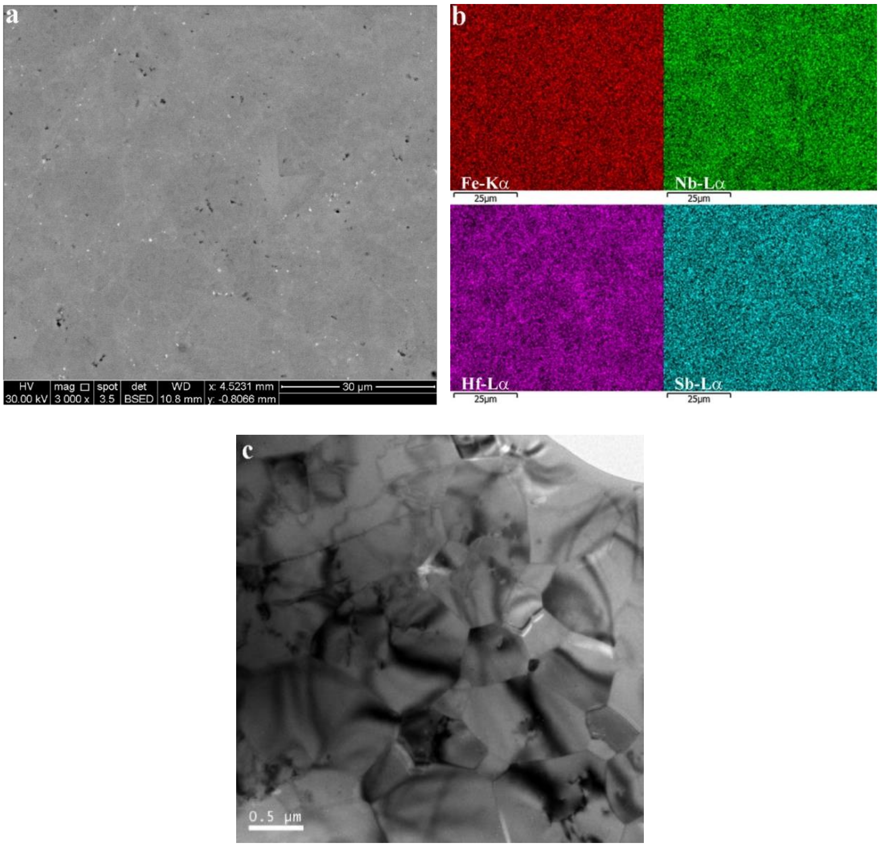

Supplementary Figure 6. SEM (**a**), EDX mapping (**b**) and TEM (**c**) images of sample $FeNb_{0.88}Hf_{0.12}Sb$.

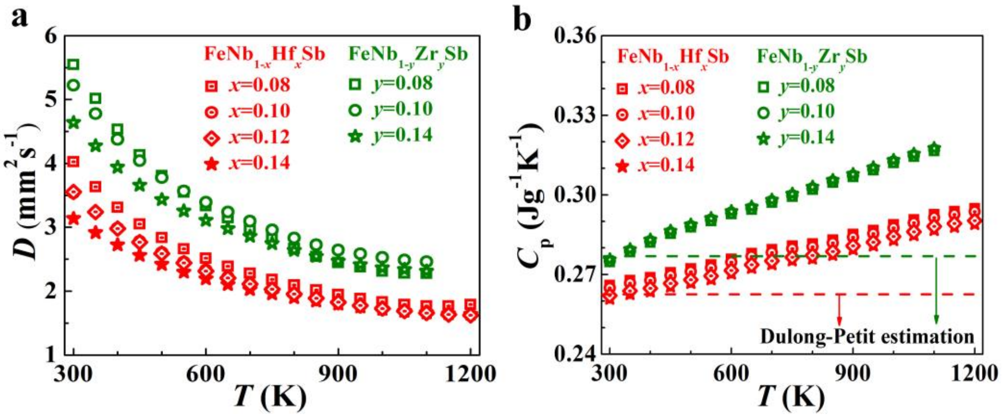

Supplementary Figure 7. Temperature dependences of thermal diffusive *D* (**a**) and specific heat *C*p (**b**) for $FeNb_{1-x}Hf_xSb$ and $FeNb_{1-y}Zr_ySb$ samples. The dash lines in (**b**) represent the Dulong-Petit estimation.

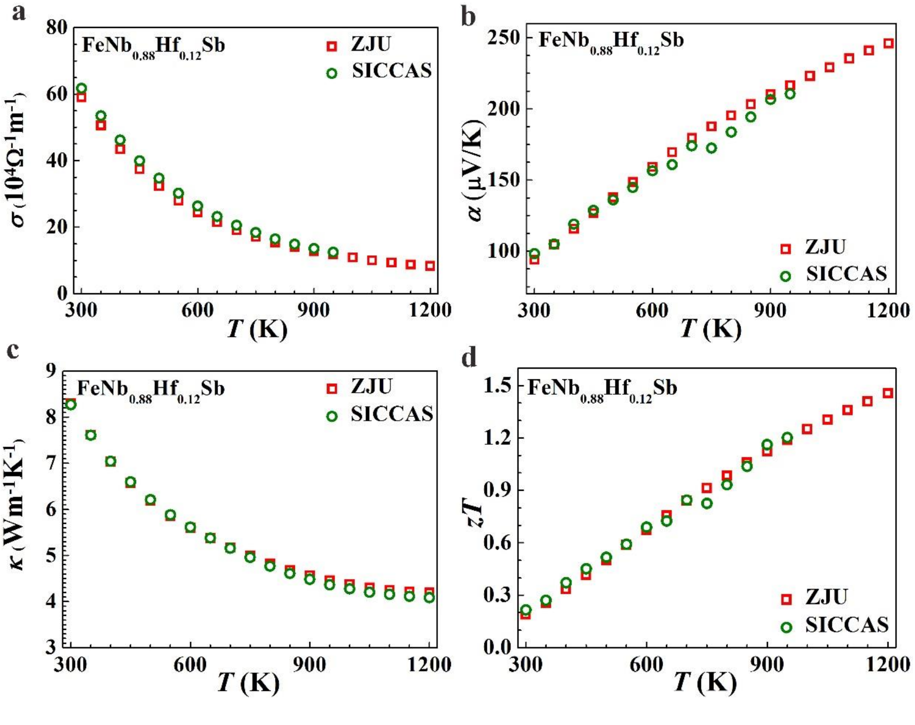

Supplementary Figure 8. The repeatability of measurement of $FeNb_{0.88}Hf_{0.12}Sb$ sample in Zhejiang University, (ZJU) and Shanghai Institute of Ceramics, Chinese Academy of Science, (SICCAS). a, electrical conductivity σ. **b**, Seebeck coefficient α. **c**, Thermal conductivity κ. **d**, zT values. The highest measurement temperature is limited by the used equipments.

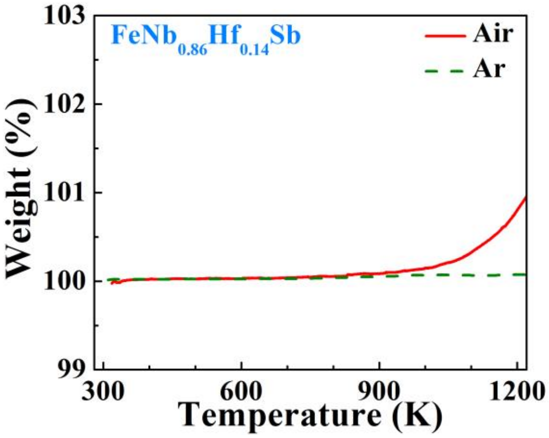

Supplementary Figure 9. Thermogravimetric analysis indicates the $FeNb_{0.86}Hf_{0.14}Sb$ compound keep stable when heating in the Ar atmosphere, while slight weight gain is found when heating in the air atmosphere above 1000K, which may result from the surface oxidation.

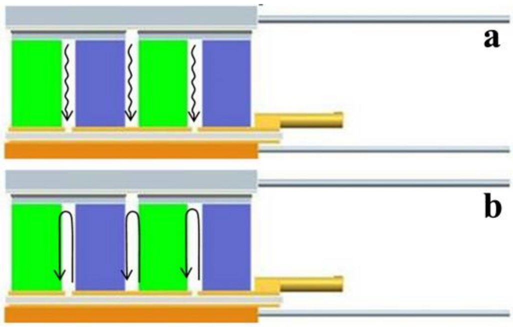

Supplementary Figure 10. The sketch map of radiation losses (a) and convection losses (b) in TE module.

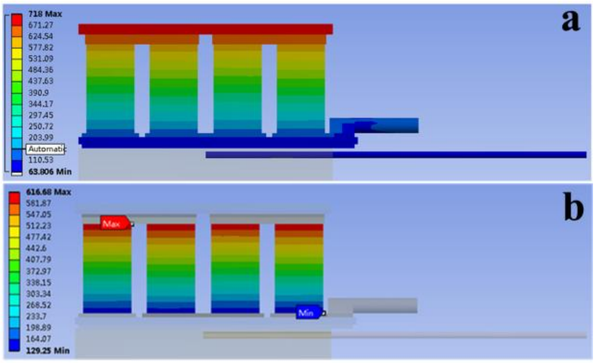

Supplementary Figure 11. Temperature distribution in TE module (a) and TE materials (b) when contact resistance taken into account.

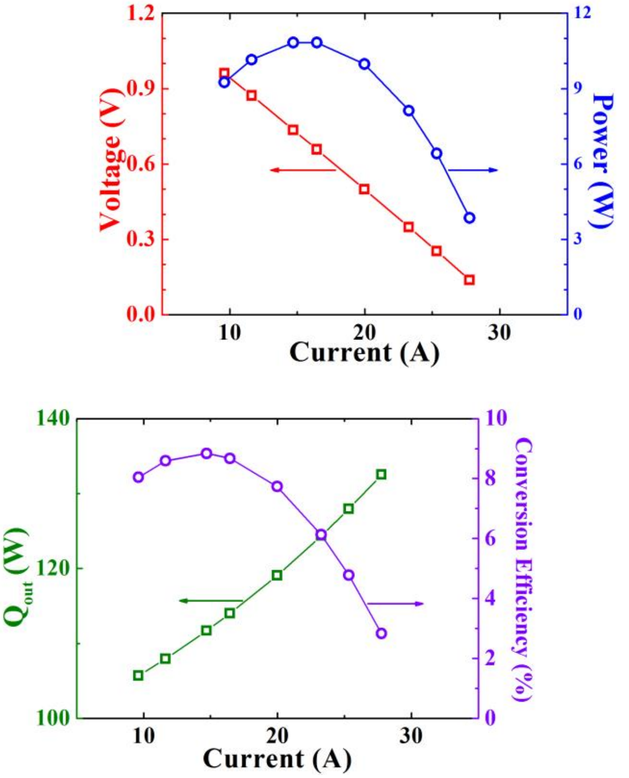

Supplementary Figure 12. Calculated TE module output properties when T_H and T_C are respectively 718 °C and 63 °C with contact resistance taken into account.

Supplementary Tables

Supplementary Table 1. Comparison of different TE modules based on half-Heusler (HH), Heusler and skutterudites compounds.

Materials	Module size (mm^3)	ΔT (K)	P_{max} (W)	Powder density (W/cm^2)
ZrNiSn/FeNbSb-based HH compounds (this work)	20×20×10	655	8.9	2.2
ZrNiSn/ZrCoSb-based HH compounds[3]	16×16×3	527	2.8	1.1
$Ti_{0.33}Zr_{0.33}Hf_{0.33}NiSn$ unileg[4]	4 times 2×2×4	565	0.044	0.275
Fe_2VAl-based Heusler compounds[5]	35×35×4.2	280	2.5	0.2
Skutterudites-based compounds[6]	50×50×7	460	11.5	0.46

Supplementary Table 2. The nominal composition and EPMA composition for $FeNb_{1-x}Hf_xSb$ and $FeNb_{1-y}Zr_ySb$ samples.

Nominal composition	EPMA composition
$FeNb_{0.94}Hf_{0.06}Sb$	$Fe_{1.01}Nb_{0.93}Hf_{0.06}Sb$
$FeNb_{0.92}Hf_{0.08}Sb$	$Fe_{1.02}Nb_{0.91}Hf_{0.08}Sb_{0.99}$
$FeNb_{0.9}Hf_{0.1}Sb$	$Fe_{1.01}Nb_{0.9}Hf_{0.09}Sb$
$FeNb_{0.88}Hf_{0.12}Sb$	$Fe_{1.01}Nb_{0.88}Hf_{0.11}Sb$
$FeNb_{0.86}Hf_{0.14}Sb$	$Fe_{1.02}Nb_{0.85}Hf_{0.14}Sb_{0.99}$
$FeNb_{0.84}Hf_{0.16}Sb$	$Fe_{1.02}Nb_{0.84}Hf_{0.15}Sb_{0.99}$
$FeNb_{0.97}Zr_{0.03}Sb$	$Fe_{1.01}Nb_{0.97}Zr_{0.03}Sb_{0.99}$
$FeNb_{0.94}Zr_{0.06}Sb$	$Fe_{1.02}Nb_{0.95}Zr_{0.05}Sb_{0.98}$
$FeNb_{0.92}Zr_{0.08}Sb$	$Fe_{1.01}Nb_{0.92}Zr_{0.07}Sb$
$FeNb_{0.9}Zr_{0.1}Sb$	$Fe_{1.01}Nb_{0.9}Zr_{0.1}Sb_{0.99}$
$FeNb_{0.86}Zr_{0.14}Sb$	$Fe_{1.02}Nb_{0.86}Zr_{0.14}Sb_{0.98}$

Supplementary Discussion

Error analysis for the discrepancy of the theoretical conversion efficiency and the experimentally determined value

The conversion efficiency of a TE module can be calculated by using the equation (1).

$$\eta = \frac{T_H - T_C}{T_H} \times \frac{\sqrt{1+ZT}-1}{\sqrt{1+ZT}+T_C/T_H} \tag{1}$$

where T_H, T_C, ZT are hot-side temperature, cold-side temperature and dimensionless of figure merit of the TE materials, respectively.

The TE module was measured using the commercial PEM-2 system. The efficiency can be calculated using equation (2), where the P_{out} and Q_{out} are the output power of the TE module and the heat flow out of the TE module, respectively.

$$\eta = \frac{P_{out}}{P_{out}+Q_{out}} \tag{2}$$

$$Q_{out} = \lambda \times \frac{(T_2 - T_3)\cdot A}{L} \tag{3}$$

The heat flow out of the TE module Q_{out} can be calculated from equation (3), where is λ, A are the thermal diffusivity of the heat sink (made of high purity copper) and the area of the heat sink (which is closed to the area of the TE module). T_2 and T_3 are the temperatures of the two different points in the middle of the heat sink. L is the distance between T_2 and T_3.

Based on the measured TE materials properties, the ANSYS software is used to simulate the temperature distribution, I-V curve, output power, and conversion

efficiency of the TE module, so that we can estimate the contribution of different effects to the reduced measured efficiency, as described below.

The HH modules in the manuscript are not filled with thermal isolation. Furthermore, the measurements are carried out under vacuum with little amount of Ar. When the module was set up in a high temperature and a large temperature difference between hot side and cold side, the convection and the radiation will take heat from the module hot side to the module cold side (See Supplementary Fig. 10). In practice, the measured heat flows Q_{out} include the heat from the TE module, the convection heat, and the radiation heat from the hot side to the cold side. In this case, the measured heat flow will higher than that of the TE module, resulting in the underestimated efficiency. Based on the equation (4), $\Phi_{H,C} \approx 7.77 / (1/\varepsilon_H + 1/\varepsilon_C - 1)$, where $\varepsilon_H = \varepsilon_C = 0.8$. The radiation loss $\Phi_{H,C}$ is about 5.18 W, corresponding to the total heat flow of 134 W. Therefore, the contribution of radiation loss to reduced efficiency is negligible, only ~0.2% underestimated efficiency. Meanwhile, the simulated convection heat loss is about 37 W, which is higher than that of radiation loss, and contributes to ~2.2% underestimation of efficiency.

$$\phi_{H,C} = \frac{A \cdot \sigma_b \cdot (T_H^4 - T_C^4)}{1/\varepsilon_H + 1/\varepsilon_C - 1} \tag{4}$$

In addition, Supplementary Fig. 11 shows the temperature distribution in the TE module when the module hot side temperature (T_{H}) and the module cold side temperature (T_{C}) are set at 718 $^{\circ}$C and 63 $^{\circ}$C, respectively. If the electrical and thermal contact resistances are not taken into account, the temperature difference in the TE materials is close to that of the TE module. The calculated theoretical

maximum output power and maximum conversion efficiency are 17.8 W and 11.3 %, respectively.

In practice, there are contact electrical and thermal resistances at the interfaces between electrode and TE materials, isolate substrate and electrode, and module and heat sink. Based on the measured contact conductivity of 20 μΩ cm^2, the calculated temperature distribution of TE materials is shown in Supplementary Fig. 11b. Actually, the contact electrical and thermal resistance will cause a reduced temperature difference across the TE materials. The real hot side temperature and the cold side temperature of the TE materials are about 616.7 ^oC and 129.3 ^oC, respectively. The actually temperature difference in TE material is only about 487.4 ^oC and it is much lower than that of ideal case (655 ^oC). Therefore, the contact electrical and thermal resistance will cause a dramatically fall of output power and conversion efficiency. The calculated maximum output power and conversion efficiency are 8.9 W and 8.1 %, respectively. (See Supplementary Fig. 12), significantly lower than the ideal case of 11.3 %. Therefore, based on the simulation results, the maximum possible contribution to the reduced conversion efficiency is poor contact properties in the TE module.

In summary, the reduced value between theoretical and experimental efficiency is due to the radiation loss, convection loss and contact resistance. The contact and convection heat loss play key roles in the reduction of the measured efficiency. Further improvement in contact and using thermal isolation in the module will significantly increase the conversion efficiency.

Supplementary References

1. Xie, H. H. *et al.* The intrinsic disorder related alloy scattering in ZrNiSn half-Heusler thermoelectric materials. *Sci. Rep.* **4,** 6888 (2014).
2. Sekimoto, T., Kurosaki, K., Muta, H. & Yamanaka, S. High-thermoelectric figure of merit realized in p-type half-Heusler compounds: $ZrCoSn_xSb_{1-x}$. *Jpn. J. Appl. Phys.* **46,** L673-L675 (2007).
3. Populoh, S., Brunko, O. C., Galazka, K., Xie, W. & Weidenkaff, A. *Materials* **6,** 1326-1332 (2013).
4. Bartholomé, K. *et al.* Thermoelectric modules based on half-Heusler materials produced in large quantities. *J. Electron. Mater.* **43,** 1775-1781 (2014).
5. Mikami, M., Kobayashi, K. & Tanaka, S. Power generation performance of thermoelectric module consisting of Sb-doped Heusler Fe_2VAl sintered alloy. *Mater. Trans.* **52,** 1546-1548 (2011).
6. Salvador, J. R. *et al.* Conversion efficiency of skutterudite-based thermoelectric modules. *Phys. Chem. Chem. Phys.* **16,** 12510-12520 (2014).
